# Technology assessment of automated atlas based segmentation in prostate bed contouring

**DOI:** 10.1186/1748-717X-6-110

**Published:** 2011-09-09

**Authors:** Jeremiah Hwee, Alexander V Louie, Stewart Gaede, Glenn Bauman, David D'Souza, Tracy Sexton, Michael Lock, Belal Ahmad, George Rodrigues

**Affiliations:** 1 Department of Epidemiology and Biostatistics, University of Western Ontario, London, Ontario, Canada; 2 Department of Radiation Oncology, London Regional Cancer Program, London, Ontario, Canada; 3 Department of Medical Biophysics, University of Western Ontario, London, Ontario, Canada

**Keywords:** radiotherapy, prostate bed, contouring, target volume delineation, contouring atlas

## Abstract

**Background:**

Prostate bed (PB) contouring is time consuming and associated with inter-observer variability. We evaluated an automated atlas-based segmentation (AABS) engine in its potential to reduce contouring time and inter-observer variability.

**Methods:**

An atlas builder (AB) manually contoured the prostate bed, rectum, left femoral head (LFH), right femoral head (RFH), bladder, and penile bulb of 75 post-prostatectomy cases to create an atlas according to the recent RTOG guidelines. 5 other Radiation Oncologists (RO) and the AABS contoured 5 new cases. A STAPLE contour for each of the 5 patients was generated. All contours were anonymized and sent back to the 5 RO to be edited as clinically necessary. All contouring times were recorded. The dice similarity coefficient (DSC) was used to evaluate the unedited- and edited- AABS and inter-observer variability among the RO. Descriptive statistics, paired t-tests and a Pearson correlation were performed. ANOVA analysis using logit transformations of DSC values was calculated to assess inter-observer variability.

**Results:**

The mean time for manual contours and AABS was 17.5- and 14.1 minutes respectively (p = 0.003). The DSC results (mean, SD) for the comparison of the unedited-AABS versus STAPLE contours for the PB (0.48, 0.17), bladder (0.67, 0.19), LFH (0.92, 0.01), RFH (0.92, 0.01), penile bulb (0.33, 0.25) and rectum (0.59, 0.11). The DSC results (mean, SD) for the comparison of the edited-AABS versus STAPLE contours for the PB (0.67, 0.19), bladder (0.88, 0.13), LFH (0.93, 0.01), RFH (0.92, 0.01), penile bulb (0.54, 0.21) and rectum (0.78, 0.12). The DSC results (mean, SD) for the comparison of the edited-AABS versus the expert panel for the PB (0.47, 0.16), bladder (0.67, 0.18), LFH (0.83, 0.18), RFH (0.83, 0.17), penile bulb (0.31, 0.23) and rectum (0.58, 0.09). The DSC results (mean, SD) for the comparison of the STAPLE contours and the 5 RO are PB (0.78, 0.15), bladder (0.96, 0.02), left femoral head (0.87, 0.19), right femoral head (0.87, 0.19), penile bulb (0.70, 0.17) and the rectum (0.89, 0.06). The ANOVA analysis suggests inter-observer variability among at least one of the 5 RO (p value = 0.002).

**Conclusion:**

The AABS tool results in a time savings, and when used to generate auto-contours for the femoral heads, bladder and rectum had superior to good spatial overlap. However, the generated auto-contours for the prostate bed and penile bulb need improvement.

## Background

Radiotherapy as an adjunct to radical prostatectomy for prostate cancer with adverse features such as pT3 and margin positive disease has established benefits of reduced disease recurrence and improved clinical outcomes [1]. Increasingly, prostate bed radiotherapy is being delivered with intensity modulated radiotherapy (IMRT) and/or image-guided radiotherapy (IGRT) which have both facilitated dose escalation to target tissues while sparing adjacent normal structures. This has improved the therapeutic ratio. However, these advanced technologies require the radiation oncologist to have a comprehensive understanding of cross sectional anatomy as compared to conventionally planned treatment (based on skeletal landmarks) for the accurate delineation and dose coverage of target volumes and organs at risk (OARs) [2]. Inadequate coverage of the prostate bed has been demonstrated to lead to an increased risk of local recurrence [3].

Significant levels of inter- and intra-observer variability in target volume delineation (TVD) has been repeatedly demonstrated in prostate cancer radiotherapy [4-7]. In fact, it has been argued that inter-observer TVD variability is the most significant contributor to uncertainty in radiation treatment planning [8]. A recent development in Radiation Oncology is the use of automated atlas-based segmentation (AABS) algorithms to aid in TVD. AABS is a computer-assisted tool that utilizes an algorithm that resamples local data to automatically outline the structures of interest to be irradiated. AABS algorithms have the potential to address the variability and time-intensive problems associated with manual contouring.

As with most technologies that are rapidly being introduced into Radiation Oncology practice, the evaluation of AABS in the form of traditional clinical trials can be costly and is likely unfeasible [9]. The purpose of this paper is to evaluate the accuracy, reliability and potential time-savings of an AABS. Secondly, we assessed inter- and intra-observer variability in the delineation of the post-prostatectomy clinical target volume (CTV) (prostate bed) and relevant organs at risk (OARs).

## Methods

Eighty post-prostatectomy patients planned for adjuvant or salvage radiotherapy from January to December 2009 were randomly selected as part of this University of Western Ontario Research Ethics Board approved study. All patients were scanned in the supine position, from L4 to the ischial tuberosities. The computed tomography (CT) images were saved according to the Digital Imaging and Communications in Medicine (DICOM) standards of practice. For all three stages of this protocol, physicians were asked to contour the prostate bed and OARs (bladder, rectum, penile bulb, bilateral femora) according to the recently published Radiation Therapy Oncology Group (RTOG) guidelines for post-prostatectomy radiotherapy [2].

**Figure 1:**
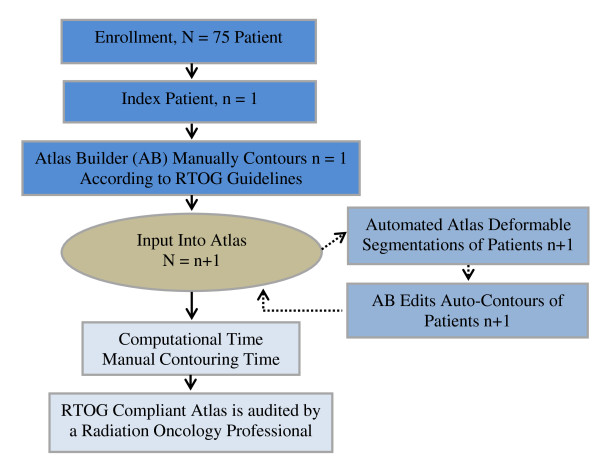
**Stage I-Atlas Building Process Map**.

In the first stage of the protocol (Figure 1), 75 patients were randomly selected to be the sample for the atlas building process. A multi-atlas segmentation approach was utilized (MIM Version 5.2, MIMVista Corp, Cleveland, Ohio) as opposed to a single-atlas segmentation approach. In a single atlas approach, only one patient is inserted into the atlas and therefore the algorithm extracts information from one subject to generate the automated contour. In a multi-atlas method, a database of pre-contoured medical images is scanned to select the most similar atlas subject based on the shape of the specified anatomical sites. Multi-atlas methods are typically used over the single atlas approach because of the improved ability to account for the large variability of anatomical regions among patients [10].

The atlas builder (GR) manually contoured an index case and inserted the contoured CT image into the atlas. A second patient was randomly selected to have the MIM atlas-based segmentation engine generate an automated contour. Since the index case was the only possible match in the atlas, the algorithm selected the index case as the best match. The pre-contoured CT image is then deformably registered onto the patient's empty CT image. The atlas pre-contoured CTV and the five OARs were warped and transformed onto the CT to create a tailored automated contour. The elapsed time for these first three steps was recorded. The auto-contour for the prostate bed, bladder, left and right femoral head, penile bulb and rectum was edited by the atlas builder according to the RTOG guidelines. The time required to edit the CTV and each of the five OARs was recorded. The final contours were then added to the atlas database, totaling two atlas subjects. The atlas builder repeated these steps for the remaining 73 patients that were selected at random. Once the atlas was completed, a second investigator (AVL) audited the final contours to ensure all contours complied with the RTOG consensus guidelines for the delineation of the prostate bed. Thus at the completion of stage I, the AABS engine had 75 reference cases with RTOG compliant segmentation for the generation of automated contours in stages II and III.

**Figure 2:**
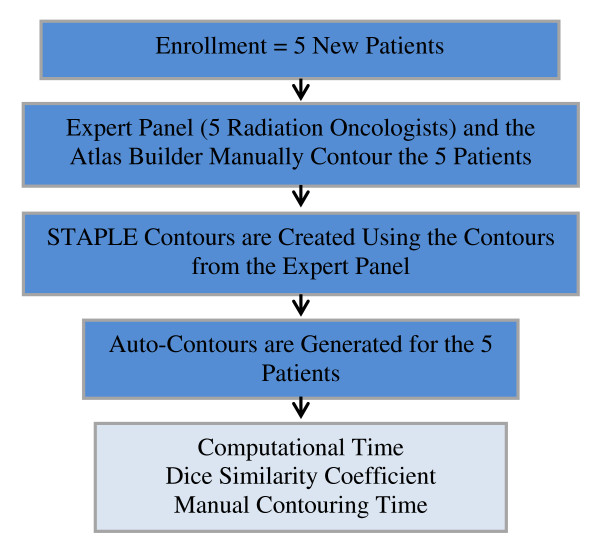
**Stage II-Assessment of the automated atlas-based segmentations and inter-observer variability**.

In stage two of the protocol (Figure 2), five Genitourinary Radiation Oncologists that routinely delineate prostate bed cases at our institution (institutional "expert panel") contoured the remaining 5 cases. Each member from the expert panel was instructed to delineate according to the RTOG guidelines and to record the total contouring time from de novo to completion. OARs were pre-labeled on the Philips Pinnacle planning system with a fixed zoom and a standardized window/level setting was applied to decrease the chance of bias and incorrect contouring. Data were gathered from the expert panel to create the simultaneous truth and performance level estimation (STAPLE) contours for each prostate bed CTV and OAR. STAPLE is an expected maximization algorithm that computes a probabilistic estimate of the true segmentation by weighing each segmentation on its estimated performance level and can be used to generate reference ("gold standard") or consensus volumes among multi-observer datasets for comparison purposes where a true gold standard may be difficult or impossible to define otherwise [11]. In parallel with creating STAPLE contours, AABS were generated for the prostate bed CTV and the five OARs in the remaining five patients. Inter-observer variability (see statistical analysis below) was assessed and baseline measurements were established to assess intra-observer variability for the third and final stage of this investigation.

**Figure 3:**
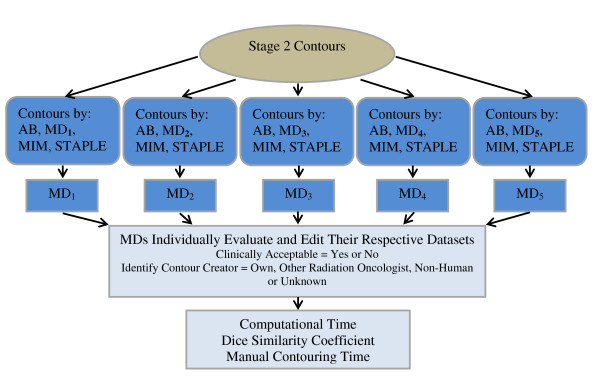
**Stage III: Validation of the automated atlas-based segmentation process**.

In stage three of this protocol (Figure 3), a set of 20 anonymized contours consisting of a strategic sample of the physician's own, the atlas builder's, AABS, and STAPLE contours (gathered from stage two) were sent to each member of the expert panel for review four weeks after the completion of stage two. They were each instructed to 1) identify the source of each contour (own, other physician, STAPLE, AABS), 2) determine if the contours were clinically acceptable or unacceptable, and 3) record the time required to edit the contours.

### Statistical Analysis

The SAS (SAS Institute Inc, North Carolina, USA) and StructSure (Standard Imaging Inc, Wisconsin, USA) were used to perform all the statistical analyses. The dice similarity coefficient (DSC) is a simple spatial overlap index that is defined as:

$$\left(V_{1},V_{2}\right)=2\left|V_{1}\cap V_{2}\left|∕\right|V_{1}\left|+\right|V_{2}\right|$$

where V_1 _and V_2 _represent the volumes of the first and the second contours respectively and ∩ is the intersection. As the DSC in contouring studies generally do not follow a normal distribution, a logit transformation was performed to allow for appropriate statistical inferences.

### Statistical Analysis Stage One: Atlas Building

Descriptive statistics and Pearson correlation coefficients were calculated to explore the performance and efficiency of the AABS tool (DSC and contour generation time as a function of number of patients in the atlas). The calculated DSC compared the initial, unedited-AABS to the version edited by the atlas builder to gain insights on the performance of the AABS. One-way quintile ANOVA assessed the contouring time in relation to the number of patients in the atlas. Shapiro-Wilk test for normal distribution was performed on the calculated DSC. Quintile ANOVA using logit(DSC) assessed the performance of the AABS engine to generate RTOG compliant segmentations for every 15 patients added to the atlas. Bonferroni correction was used to adjust for multiplicity in the quintile comparisons.

### Statistical Analysis Stage Two: Assessment of the AABS and Inter-observer Variability

The DSC was calculated to compare the AABS, expert panel members ("observers") and the atlas builder. Descriptive statistics were calculated to illustrate overall inter-observer variability. Shapiro-Wilk test for normal distribution was performed on the calculated DSC. One-way analysis of variance was performed using logit(DSC) to test for inter-observer variability in the delineation of the CTV and five OARs among the expert panel. Two-way analysis of variance was performed modeling the effects of the observer and patient on logit(DSC) values for the CTV and five OARs, and the effects of the observer and patient on the contouring time.

### Statistical Analysis Stage Three: Validation of the Automated Atlas-Based Segmentations

The DSC was calculated for a number of spatial overlap comparisons to determine the convergence of the edited automated contours towards the gold standard and intra-observer variability in the delineation of the CTV and five OARs. Descriptive statistics was calculated to describe the performance of the AABS engine using DSC and the total contouring time for human observers and non-human raters. A paired t-test was performed to assess differences in the time required to edit the auto-contours and the de novo manual contouring time.

### Attempts to Minimize Bias

Four measures were taken to minimize bias. The first attempt to eliminate bias occurred at the construction of the RTOG atlas stage through the appraisal of the edited contours by a second radiation oncology expert to ensure compliance. Calculating the DSC between the atlas builder and STAPLE generated consensus contours at stage two to evaluate the appropriateness of that particular radiation oncologist as the atlas builder was the second attempt to minimize bias. Sending the expert panel the anonymized blinded dataset to be assessed in stage three was used as another attempt to minimize bias. In stage three the expert panel was blinded as to the source of the contours in assessing intra-observer variability to hopefully prevent any bias the expert panel may have had if they knew the creator of the contour. Finally, waiting four weeks after the expert panel finished stage two before sending the anonymized data set to the expert panel to be reviewed was designed to prevent the members from recalling their own contours.

## Results

### Stage I

In stage one, generating AABS for the 75 patients took an average of 108 seconds per patient (standard deviation, SD = 25 seconds, range 68 to 200 seconds). ANOVA suggested no improvements in auto-contouring time as the number of subjects increased in the atlas (p value = 0.28). The mean (SD) for the auto-contouring time for quintile 1, 2, 3, 4, and 5 were 103 (37), 97 (11), 109 (27), 114 (23) and 115 (22) seconds, respectively (p = 0.282 between quintiles).

The mean (SD) time for the atlas builder to edit the automated contours were: 154 seconds (71 seconds) for the prostate bed, 156 seconds (79 seconds) for the bladder, 125 seconds (80 seconds) for the left femoral head, 97 seconds (61 seconds) for the right femoral head, 19 seconds (9 seconds) for the penile bulb and 149 seconds (65 seconds) for the rectum. The DSC was calculated to compare the edited auto-contours by the atlas builder to the initial auto-contours generated by the AABS tool. The mean (SD) DSC for the CTV and the OARs was 0.65 (0.16) for the prostate bed, 0.73 (0.18) for the bladder, 0.95 (0.04) for the left femoral head, 0.96 (0.04) for the right femoral head, 0.60 (0.28) for the penile bulb and 0.68 (0.13) for the rectum. Table 1 illustrates the descriptive statistics for the quintile analysis (n = 15 per group) for every 15 patients added to the atlas for each OAR and the CTV to evaluate the performance of the AABS as more subjects are added.

**Table 1:** The ability of the automated atlas-based segmentation tool to generate segmentations compliant with the consensus guidelines as more subjects are added to the atlas

Variables	Quintile 1	Quintile 2	Quintile 3	Quintile 4	Quintile 5
DSC mean (SD)					
Prostate Bed	0.63 (0.13)	0.64 (0.20)	0.63 (0.17)	0.71 (0.13)	0.66 (0.16)
Bladder	0.58 (0.15)	0.75 (0.16)	0.74 (0.20)	0.84 (0.10)	0.72 (0.17)
LFH	0.90 (0.07)	0.96 (0.02)	0.96 (0.02)	0.97 (0.02)	0.97 (0.02)
RFH	0.93 (0.04)	0.94 (0.04)	0.97 (0.01)	0.97 (0.01)	0.96 (0.04)
Penile Bulb	0.37 (0.39)	0.65 (0.23)	0.60 (0.27)	0.72 (0.13)	0.64 (0.23)
Rectum	0.62 (0.12)	0.72 (0.15)	0.66 (0.15)	0.71 (0.13)	0.68 (0.11)

DSC = dice similarity coefficient.

SD = standard deviation.

LFH = left femoral head.

RFH = right femoral head.

### Stage II

**Figure 4:**
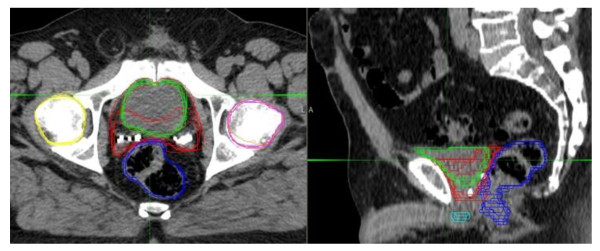
**Axial and Sagittal Computed Tomography Image Demonstrating Individual Contours From the Expert Panel**. Colors: red represents the contours for the prostate bed; green represents the contours for the bladder; pink represents the contours for the left femoral head; yellow represents the contours for the right femoral head; royal blue represents the contours for the rectum; and teal represents the contours for the penile bulb.

In stage two, five new subjects were used to test the performance of the atlas and inter-observer variability (Figure 4). Table 2 illustrates the DSCs evaluating the MIM generated auto-contours against STAPLE (estimated truth) and the expert panel as well as inter-observer variability among the Radiation Oncologists. The MIM AABS tool had higher mean DSC when compared to the STAPLE than compared to the observers for the CTV and all OAR. The variability in the DSC seen in the comparisons between the auto-contours versus STAPLE and the auto-contours versus the expert panel for the prostate bed, bladder, penile bulb and the rectum regions are comparable.

**Table 2:** DSCs of the CTV and ROIs, assessing auto-contours and inter-observer variability

Variables	AC vs. STAPLE	Edited AC vs. STAPLE	AC vs. Expert Panel	STAPLE vs. Expert Panel	Observers vs. Other Observers	AB vs. STAPLE
Prostate Bed	0.48 (0.17, 0.18-0.59)	0.67 (0.19, 0.18-0.91)	0.47 (0.16, 0.11-0.64)	0.78 (0.15, 0.37-0.91)	0.65 (0.14, 0.29-0.84)	0.93 (0.03, 0.90-0.96)
Bladder	0.67 (0.19, 0.34-0.80)	0.88 (0.13, 0.34-0.97)	0.67 (0.18, 0.33-0.81)	0.96 (0.02, 0.92-0.98)	0.94 (0.03, 0.87-0.97)	0.97 (0.01, 0.95-0.99)
Left Femoral Head	0.92 (0.01, 0.92-0.93)	0.93 (0.01, 0.92-0.97)	0.83 (0.18, 0.43-0.93)	0.87 (0.19, 0.47-0.98)	0.76 (0.23, 0.42-0.99)	0.96 (0.01, 0.95-0.98)
Right Femoral Head	0.92 (0.01, 0.91-0.93)	0.92 (0.01, 0.90-0.96)	0.83 (0.17, 0.45-0.94)	0.87 (0.19, 0.46-0.98)	0.77 (0.23, 0.46-0.99)	0.97 (0.01, 0.95-0.98)
Penile Bulb	0.33 (0.25, 0.10-0.70)	0.54 (0.21, 0.10-0.78)	0.31 (0.23, 0-0.78)	0.70 (0.17, 0-0.88)	0.55 (0.22, 0-0.84)	0.84 (0.07, 0.75-0.94)
Rectum	0.59 (0.11, 0.48-0.77)	0.78 (0.12, 0.49-0.90)	0.58 (0.09, 0.45-0.77)	0.89 (0.06, 0.67-0.94)	0.83 (0.07, 0.65-0.91)	0.94 (0.02, 0.92-0.96)

Mean DSC (SD, Range)

AC = auto-contours

STAPLE = Simultaneous Truth and Performance Level Estimation

AB = atlas builder

DSC = dice similarity coefficient

SD = standard deviation

The spatial overlap between the atlas builder and STAPLE was calculated to determine if the atlas builder contours the CTV and ROI as the community of radiation oncologists would contour these regions. The mean DSC (SD, range) was 0.93 (0.03, 0.90-0.96) for the prostate bed, 0.97 (0.01, 0.95-0.99) for the bladder, 0.96 (0.01, 0.95-0.98) for the left femoral head, 0.97 (0.01, 0.95-0.98) for the right femoral head, 0.84 (0.07, 0.75-0.94) for the penile bulb and 0.94 (0.02, 0.92-0.96) for the rectum.

One-way ANOVA on DSC between Radiation Oncologists was performed to evaluate inter-observer variability. At least one observer significantly differed from the other observers when contouring the prostate bed (p value = 0.002), left femoral head (p value < 0.001) and right femoral head (p value < 0.001). There was no significant difference among observers when contouring the bladder, penile bulb and the rectum. Two-way ANOVA modeling the effects of the observer and patient on the DSC was performed. This revealed significant differences in the delineation of the prostate bed, (p < 0.001). Observer and patient differences significantly predicted for variability in DSC for prostate bed (p < 0.001, p = 0.006) and bladder (p = 0.002, p < 0.001). Variability in right and left femoral heads DSC was significantly dependent on the observer only (both p < 0.001), while variability in rectum and penile bulb delineation was dependent on patient factors (p < 0.001). Another two-way ANOVA analysis modeling the effects of the observers and patients on the contouring time was performed. The full two-way model for the contouring time was significant (p < 0.001) with both the observers (p < 0.001) and the patients (p < 0.001) having a significant effect on the contouring time.

### Stage III

With regards to stage three, Table 2 displays the results of the DSC comparing the edited-auto-contours by the expert panel to the STAPLE. The highest spatial overlap was seen in the left femoral head and the right femoral head, while the lowest spatial overlap was seen in the penile bulb. The second lowest spatial overlap was seen in the prostate bed. These results are consistent with those seen in stage two that compared the unedited auto-contours to STAPLE.

The expert panel was sent an anonymized representative contour sets generated by another expert panel member, the AABS, the STAPLE algorithm. Prior to any editing of the stage two contours by the observers, the observers were asked if the contours were acceptable. Of the 100 cases distributed, 78% of the human contours, 96% of the STAPLE contours, and 12% of the MIM auto-contours were considered clinically acceptable. The expert panel was also asked to identify the source of the contours. Out of the 50 non-human contours, 54% were correctly identified while out of the 50 human contours, 70% was correctly identified. The probability that a Radiation Oncologist was able to properly identify his own contours was 56%.

The panel members were asked to edit the contours as clinically necessary. There appeared to be little intra-observer variability among the edited contours among the expert panel. The penile bulb had the lowest mean DSC at 0.89 (0.04, 0.84-0.98) which is still considered to be good spatial overlap. The remaining OARs intra-observer variability DSC were: prostate bed 0.94 (0.04, 0.84-0.98), bladder 0.98 (0.01, 0.96-0.99), left femoral head 0.97 (0.01, 0.96-0.99), right femoral head 0.97 (0.01, 0.95-0.99), and rectum 0.94 (0.04, 0.80-0.98).

The mean (SD) contouring time for all five cases for the edited auto-contouring time and the manual contouring time was 14.1 minutes (8.4 minutes) and 17.5 minutes (5.4 minutes) respectively, equating to an average 24% time reduction when using the AABS tool. A paired t-test comparing the times of the edited auto-contouring to the manual contouring time showed significant difference in contouring times (p value = 0.003).

## Discussion

Inter-observer variability in segmentation (targets and organs at risk) may be the most significant contributor to uncertainty in radiation treatment planning [8]. We have shown that even with the use of consensus guidelines, inter-observer variability still exists. With these findings, it is important to continue to address the variability challenges. Computerized contouring aids can potentially reduce this variability and increase efficiency in the segmentation workflow and AABS is one such tool. This was the first study to evaluate automated atlas based segmentations for the prostate bed. In this study we evaluated and validated contours created by atlas-based segmentation engines in the context of segmentation of post-prostatectomy radiotherapy planning CT datasets. In the context of this study, only 12% of the unedited contours generated by the AABS were found to be clinically acceptable by the expert panel. Specifically, while the AABS tool appears to reasonably delineate the femoral heads, bladder and rectum, the delineation of the prostate bed and penile bulb were unacceptable. The edited-auto-contours for the femoral heads, bladder and rectum had superior to good spatial overlap when compared to the gold standard. However, the edited-auto-contours for the prostate bed and the penile bulb require improvement when compared to the gold standard. The penile bulb represents a small volume, and thus small variations in its contouring will result in a large change in DSC. In terms of the prostate bed our findings are not surprising given that AABS algorithms are typically developed to detect and segment intact structures and the prostate bed is a "virtual" target defined by boundaries of surrounding normal tissues based on known patterns of recurrence and expert opinion rather than a discrete structure.

The inherent difficulty in defining the "virtual" prostate bed target is reflected in the presence of inter-observer variability in the delineation of the prostate bed and has been repeatedly demonstrated in the literature [2-4,12,13]. This variability appears to persist even despite the use of rigorous contouring protocols and guidelines [12,14-16]. Symon *et al*., in their study of prostate bed contouring variability, defined a high-risk volume, which on average is missed in 27.5% (range, 2.3%-78.7%) of cases. At least 25% of the high-risk volume at the bladder neck anastomosis and the retro-vesical space was excluded in 11 out of 38 CTVs [13]. Our study found that intra-observer variability was a smaller source of TVD error than inter-observer variability, consistent with the literature [3,17]. Wiltshire *et al*. quantified TVD variability using a distance-based approach, and found consistent inter-observer variability within the anterioposterior and superioinferior dimensions measuring a mean (SD) distance between contours of 3.8 mm (2.2 mm) and 1.2 mm (2.3 mm) respectively. The main source of the intra-observer variability in this study was in the anterior-posterior dimension measuring a mean (SD) distance between contours of 0.4 mm (1.2 mm).

The use of AABS tools to delineate OARs for other cancer disease sites including head and neck [10], breast [18], and endometrium [19] have been shown to reduce TVD variability and the total time required to contour; in our study the main benefit of the AABS was in decreasing the amount of time for contouring through editing of the auto contours rather than requiring de novo generation of contours.

The conclusions of this study need to be considered in the context of its limitations. The 80 post-prostatectomy cases used from our institution may limit the applicability of the atlas to other practice groups. Incorporating all available patients into the atlas building process does have a drawback. While increasing the number of patients added to the atlas increases the potential to account for differences in anatomy post surgery, it is at the cost of computational time. The larger the atlas, the longer it will take the tool to search through the atlas to select the best match. Other studies used 10 patients [10] and one study that assessed the same AABS tool included 15 patients in their atlas [19]. We found no improvement in performance of the AABS when analyzed by quintile; suggesting a dataset of 15 patients may be sufficient to provide auto contours that are useful for subsequent editing/refinement.

This study's methodology builds on the available literature to improve the methodological strength. The strengths of the methodology include the use of consensus guidelines, anonymized datasets, the blinding of observers, the creation of a ground truth, and our specific measures to limit bias, especially with the comparison of the atlas builder to the ground truth. Except for our attempts to limit bias, this methodology is similar to that used in another study [20]. The differences are in the attempts to limit bias and the statistical analyses.

We recommend that the MIM AABS tool can be adopted for routine clinical use to generate auto-contours for the bilateral femoral heads with no editing required. For the bladder and rectum, the auto-contours require some editing by a Radiation Oncologist. Clinical use of the atlas requires a Radiation Oncologist to review and edit the auto-contours, in particular for OARs where the AABS underperforms such as the penile bulb and prostate bed CTV. The automated contouring workflow from a clinical perspective was shown to be significantly shorter than the manual contouring process. The methodology highlights the strengths and areas of improvement for AABS and systematically assesses the presence and amount of inter- and intra-observer variability. If contouring practices for CTVs and OARs converge with the adoption of contouring guidelines, AABS algorithms may be programmed in parallel with these guidelines to optimize how Radiation Oncologists delineate targets. Performing these tasks in a systematic manner through technological assessment as demonstrated in this paper is crucial to ensure the appropriate use of such tools in clinical practice. As the field of AABS advances, it becomes increasingly important to evaluate the accuracy and reliability of the atlas-based segmentations to garner empirical evidence to support the decision-making process prior to its adoption for routine clinical use.

## Competing interests

The authors declare that they have no competing interests.

## Authors' contributions

JH drafted the manuscript and performed the statistical calculations. AL coordinated participation in the study and assisted in manuscript preparation and drafting. GB, TS, DD, ML, and BA participated in the study. GR and SG conceived and coordinated the design of the study. All authors read and approved the final manuscript.
